# Beyond eruptive scenarios: assessing tephra fallout hazard from Neapolitan volcanoes

**DOI:** 10.1038/srep24271

**Published:** 2016-04-12

**Authors:** Laura Sandri, Antonio Costa, Jacopo Selva, Roberto Tonini, Giovanni Macedonio, Arnau Folch, Roberto Sulpizio

**Affiliations:** 1 Istituto Nazionale di Geofisica e Vulcanologia, Sezione di Bologna, Bologna, Italy; 2 Istituto Nazionale di Geofisica e Vulcanologia, Sezione di Roma1, Roma, Italy; 3 Istituto Nazionale di Geofisica e Vulcanologia, Osservatorio Vesuviano, Napoli, Italy; 4 Barcelona Supercomputing Centre, Barcelona, Spain; 5 Università di Bari, Dipartimento di Scienze della Terra e Geoambientali, Bari, Italy

## Abstract

Assessment of volcanic hazards is necessary for risk mitigation. Typically, hazard assessment is based on one or a few, subjectively chosen representative eruptive scenarios, which use a specific combination of eruptive sizes and intensities to represent a particular size class of eruption. While such eruptive scenarios use a range of representative members to capture a range of eruptive sizes and intensities in order to reflect a wider size class, a scenario approach neglects to account for the intrinsic variability of volcanic eruptions, and implicitly assumes that inter-class size variability (i.e. size difference between different eruptive size classes) dominates over intra-class size variability (i.e. size difference within an eruptive size class), the latter of which is treated as negligible. So far, no quantitative study has been undertaken to verify such an assumption. Here, we adopt a novel Probabilistic Volcanic Hazard Analysis (PVHA) strategy, which accounts for intrinsic eruptive variabilities, to quantify the tephra fallout hazard in the Campania area. We compare the results of the new probabilistic approach with the classical scenario approach. The results allow for determining whether a simplified scenario approach can be considered valid, and for quantifying the bias which arises when full variability is not accounted for.

Volcanic hazard assessment has been one of the most pursued goals in volcanology^1^,^2^,^3^,^4^, especially for volcanoes near densely inhabited areas such as the volcanoes in the Neapolitan region [e.g.^5^,^6^], where about three millions people are potentially exposed to tephra fallout. Pioneer studies^7^ focused on the mapping of deposits of past eruptions, under the principle that past is the key to future. More recent works, starting from the reconstruction of size and dynamics of past eruptions, introduced the concept of “eruptive scenario”, a multifaceted term indicating a broad set of eruptive conditions. For example, the first tephra fallout probability maps for the Neapolitan area^2^ which accounted for wind variability were produced using eruption of a particular size and intensity (i.e., a scenario, characterised by a total erupted mass or volume, and a column height or mass eruption rate respectively), on the basis of time-predictable behaviour of Somma-Vesuvius^8^. In this framework, for a specific total erupted mass, the effects of variable column heights and duration were first considered^9^. More recently, the modelling of tephra fallout hazard was computed as the mean hazard over many simulations in which volcanological parameters were randomly sampled from suitable ranges^10^,^11^. This method was named “eruption range scenario” and its extension, where several eruptive events are simulated, the “multiple eruption scenario”. This approach succeeds in partially quantifying natural variability but is limited to consideration of only a specific range of eruptive magnitudes.

In recent times, the paramount importance of quantifying uncertainty has been recognised in volcanic hazard assessment. When assuming a scenario, we are implicitly neglecting a large set of uncertainties, both aleatory, which reflects the intrinsic natural variability of eruptive processes, and epistemic, due to our limited knowledge on such processes and to our simplifications in trying to model them^12^.

Recent literature on tephra fallout hazard assessment^5^,^13^,^14^,^15^,^16^ accounts for eruptive size and intensity variability by assuming a few “representative eruptive scenarios”, where one scenario is used to represent a sub-range of eruptive sizes, intensities, and vent position. By means of a Bayesian Event Tree model^17^, the effects of natural variability, in terms of both eruptive sizes and vent position, have been investigated in detail with respect to the tephra fallout probability maps at Campi Flegrei^16^. In particular, that study explored the combined effect of accounting for a range of wind profiles, vent positions, and four different eruptive sizes (three explosive and one effusive). While the spatial uncertainty of the vent position was taken into account by considering several (700) potential vent positions across the caldera, the four eruptive sizes were chosen based on volcanological parameters which characterised four specific past events of the Campi Flegrei, which we call *representative members*. These representative members were assumed to be representative for the whole spectrum of possible eruptive magnitudes and intensities of potential future events at this volcano. The study showed that vent position represents a significant source of uncertainty given the dimensions of the caldera. The definition of representative scenarios, and in particular of representative members, implies a more or less subjective choice of the parameters characterising the “representative eruptions”. Making such a choice implies that a discrete number of representative members are able to describe the whole natural variability. The main assumption behind this discretization is that the variability due to combinations of parameters characterising eruptions belonging to the same eruptive class (intra-class variability) is negligible when compared to the inter-classes (inter-members) variability. While this discretization is motivated by an effort to simplify and reduce computational efforts, it has never been quantified whether or not such an assumption has a substantial effect on the final hazard assessment.

In this paper we introduce a novel approach for sampling and weighting possible statistical combinations of values for the volcanological parameters. The weights of these different combinations correspond to their probability of occurrence (as in all PVHA): this enables giving more weight to more likely combinations. In particular, our general strategy follows these steps:

- We select a very broad range of possible eruptive sizes identified by the total erupted mass; the total erupted mass is used to define the eruption magnitude^18^;
- We split the eruptive size range into a few classes that can be linked to representative members similar to the classical approach used in past studies. These classes ideally span the general range continuosly, whereas representative members, by definition, discretize it;
- Within each class, we randomly sample the crucial input parameters of tephra dispersal simulators, such as the total erupted mass, the fraction of mass associated with the tephra fallout phase, and the duration of the fallout phase, covering all the plausible values. The mass eruption rate of the fallout phase is obtained by dividing the mass erupted in such phase by its duration. The other pivotal parameters of the tephra dispersal simulators are estimated in a consistent manner, so that all the eruptive features are coherent with what retrieved from the tephra deposits of representative members and similar eruptions. In particular, eruptive column height values are chosen consistently with the mass eruption rates, and total grain size distribution with field data relative to deposits of similar eruptions. This allows us to explore the effect of the parameters’ variability *within* each class, without losing the consistency among strongly related parameters;
- We assume a power law distribution for the total erupted mass, evaluating the probability of each combination of controlling parameters. The assumption of a power law for the total erupted mass (cf. also in refs 19,20) allows a smooth and coherent linking of the different classes into a total probability distribution (similarly to earthquake frequency-size probability distribution commonly known as the Gutenberg-Richter law^21^).

The effects of this new method on uncertainty quantification are explored using a Bayesian Event Tree model^17^ that incorporates epistemic uncertainty. The method is applied to the two main volcanic systems threatening the Neapolitan area, *i.e.*, Somma-Vesuvius and Campi Flegrei (see Fig. 1). These volcanoes have been selected for the large amount of past studies aimed at quantifying the impact of tephra fallout in case of a renewal of their activity. This enables a full comparison between the maps for tephra fallout obtained with this novel methodology, and the classical ones based on representative members (*i.e.*^14^,^16^ for Campi Flegrei and Somma-Vesuvius respectively). Tephra load is a common intensity measure of the hazard associated to tephra fallout, as it expresses the mass of tephra accumulated at the ground (or on roofs etc.) for unit area (typical unit is kg/m^2^). In this paper, we will compare *hazard maps* and *probability maps*. As commonly used in the literature (e.g.^22^), we use the term “hazard map” to indicate a map showing, for every grid point, the tephra load having a probability equal to a selected value of being overcome. Similarly, a “probability map” shows, for every grid point, the probability to observe a tephra load equal to, or larger than, a specific threshold. The term “conditional map” means that the map displays the results “in case of an eruption from the vent and of the size specified”.

For modelling tephra dispersal we use to different solvers, the analytical simulator HAZMAP^23^ and the numerical simulator FALL3D^24^. The results of the simulations are first compared, and then used to quantify differences due to the two different methods (the new and the classical ones). The use of two different simulators (*i.e.*, HAZMAP and FALL3D) allows us to demonstrate the applicability of the proposed approach with multiple simulators and its potential for future applications considering model ensembles.

Besides the scientific importance of understanding implications of the parameter space discretization, the results we obtain can quantify the subjective assumptions made in volcanic hazard assessment in order to reduce the computational efforts by simplifying the natural variability considering only a few representative members.

## Results

Figure 2 shows the Probability Density Functions (PDF) of total erupted mass, for Vesuvius and Campi Flegrei separately, that we built (see Methods) in order to assign a weight to each simulation run; in this way, within each class, we give a higher weight to the output of more likely (*i.e.*, smaller magnitude) simulated eruptions, and so we account for the intra-size variability.

We have built these PDF from previous studies^25^,^26^ on the relative probability of the different possible explosive size classes, conditional to eruption occurrence at Somma-Vesuvius and Campi Flegrei, respectively. Remarkably, although the criteria to set the PDF parameters do not pose any constraint on continuity among the three eruptive size ranges, the result is an almost continuous relationship, linking them smoothly. The PDF are then used to assign a conditional probability of occurrence to each simulation as a function of the associated eruption magnitude.

**Figure 1:**
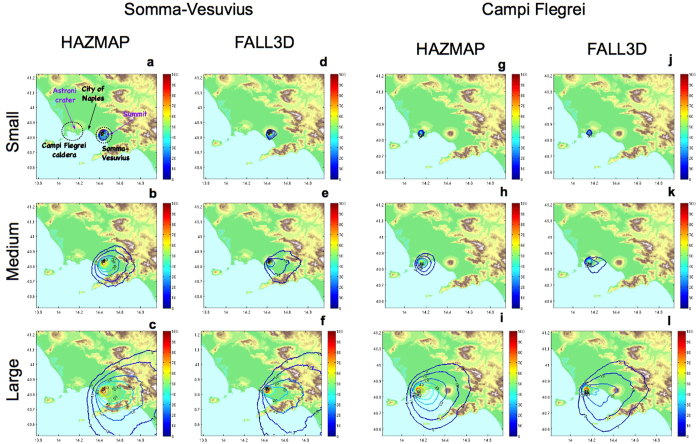
Panel (**a**): Map of the Neapolitan area, showing the location of Somma-Vesuvius and Campi Flegrei caldera with respect to the city of Naples. The location of the most likely vent positions (Astroni for Campi Flegrei^41^, and the summit area for Somma-Vesuvius^42^) are shown. All panels: Comparison between results with the new approach at Somma-Vesuvius and Campi Flegrei, as achieved on from simulations performed by HAZMAP (panels (**a–c**) and (**g–i**) respectively) and by FALL3D (panels (**d–f**) and (**j–l**) respectively). Results are given in terms of probability of overcoming 300 kg/m^2^, given an eruption of a given size from the summit vent at Somma-Vesuvius, or Astroni vent at Campi Flegrei, as specified. Background maps in this figure have been generated by using the Shuttle Radar Topography Mission (SRTM) data set (90 × 90 m) provided by the CGIAR Consortium for Spatial Information (http://srtm.csi.cgiar.org/) and they have been incorporated in the corresponding panel using Python (https://www.python.org/) software (including Numpy (http://www.numpy.org), and Matplotlib (http://matplotlib.org/) packages); panels have been assembled with LibreOffice (https://www.libreoffice.org/).

First, we compare the results obtained by applying the new method using the simulations of the two different tephra dispersal simulators, *i.e.*, HAZMAP and FALL3D. In Fig. 1 we show the conditional probability maps for a given vent (summit for Somma-Vesuvius, Astroni for Campi Flegrei) and size class (Small, Medium, and Large) for a tephra load of 300 kg/m^2^. We see that the patterns in the isoprobability contours are very similar for both simulators, except for a larger deposit load in HAZMAP simulations at proximal distances, likely due to the different description of eruption column in the two simulators (the HAZMAP column model does not account for wind effects, while in FALL3D we explicitly consider air entrainment and bending of the column due to wind). For the sake of conciseness, hereinafter we focus on the comparison between the classical and new approach analysing the HAZMAP simulations only. This choice is also dictated by the fact that the results obtained with the classical method were also obtained using HAZMAP^14^,^16^.

In the following, we will call the hazard models obtained through representative members^14^,^16^ as *classical models*, while the methodology on which they are based will be termed as *classical method*.

In the context of comparing our results with previous works relying on representative members, we recalculate the classical method map^14^,^16^ at Somma-Vesuvius and Campi Flegrei. Then, we compute three different types of comparisons:

1. Maps reporting the difference in the conditional probability maps (the new and the classical ones) of overcoming a tephra load threshold of 300 kg/m^2^;
2. Maps showing the difference in the conditional *hazard maps* (the new and the classical ones) of the mean tephra load with an exceedance probability of 5%;
3. A comparison, as in ref. 27, of the area enclosed by the 5% isoprobability contour line of exceeding different thresholds in tephra load (from now on, the “hazard area”) between our conditional probability maps (from HAZMAP and FALL3D simulations) and the one from the classical model.

Comparisons between the results obtained with the new and classical methods are made based on the mean values resulting from all simulations.

The comparisons analyse three different cases:

1. First of all, we separately analyse the different eruptive size class range, keeping fixed the eruptive vent in the most likely position for each volcano (summit area for Somma-Vesuvius, Astroni for Campi Flegrei). In Figs 3 and 4 we show the difference in probability maps (a,b,c), in hazard maps (d,e,f), and hazard areas (g,h,i) according to the eruptive size class, for the two volcanoes respectively. These figures show the results obtained with the HAZMAP simulator (using FALL3D produces very similar results).
2. For both volcanoes, we analyse also the differences between the new and classical methods when accounting for all the possible eruptive sizes, given an eruption from the most likely vent position^16^ (again summit area for Somma-Vesuvius, Astroni for Campi Flegrei). The differences in probability maps (a,b), in hazard maps (c,d), in the hazard areas (e,f) are shown in Fig. 5.
3. For Campi Flegrei, we also explore the effect of the vent position variability, first by showing the comparison of the results obtained combining all possible vent positions given an eruptive size, and then by combining all the possible size classes from any possible vent^16^,^28^ conditional probability to the occurrence of an eruption, *i.e.*, considering the uncertainty on both the size class and vent position. The differences in probability maps (a, d, g, j), in hazard maps (b, e, h, k), in the hazard areas (c, f, i, l) for the new and the classical methods are shown in Fig. 6.

## Discussion

The goal of this paper is to quantify how the more or less subjective choice of a few representative eruptions, at the basis of the classical method for probabilistic tephra fallout hazard assessment, influences hazard and probability maps of tephra fallout. As making accurate forecast of natural phenomena is the only convincing evidence that science is really improving our knowledge^29^,^30^, it is of paramount importance to formulate robust PVHA models, and to identify and quantify potential biases introduced by assumptions and simplifications. To this end, we propose an innovative method to explore the intra-class variability and to weight each possible combination of values of the eruptive parameters in a PVHA perspective. Then, we quantify the relative difference in the probability maps obtained with this new method, compared to the classical method based on representative members. We also quantify the difference in the hazard maps, as well as changes in the area enclosed by the 5% isoprobability contour line, for different tephra load thresholds.

Concerning the use of multiple models, we first check that the results, in terms of probability maps, are not substantially different when using different simulators (Fig. 1). The influence of the simulator on the statistical result is relatively little, with results obtained with the analytical simulator HAZMAP and the numerical simulator FALL3D very similar. The main relevant difference between the results of the two simulators is in the proximal hazard area, which is generally larger for the maps made with HAZMAP for different loads. Nonetheless, we want also to stress the general applicability of the new method to deal with multiple simulators and hence, potentially with an ensemble of models. Future research should be devoted to characterising such differences in connection to the epistemic uncertainty quantification that Bayesian methods enable and should focus on the analysis of the results when the new approach is applied to the results from an ensemble model^31^,^32^.

Keeping the eruptive vent position fixed at the most likely location for each volcano, and looking at the different eruptive size classes separately (Fig. 3 for Somma-Vesuvius and Fig. 4 for Campi Flegrei), we can see that the classical method tends to produce a larger estimate of hazard (both in terms of probability and hazard maps) in proximal areas for both volcanoes. On the other hand, when considering the Medium and especially the Large size class, the classical method produces a widespread underestimation in medium to distal areas (10 to 30 km downwind), up to 10–20% in probability, and approximately up to 500 kg/m^2^ in the mean tephra load. This effect is more evident for Somma-Vesuvius, and it persists even when considering the combination of simulations accounting for all the possible eruptive size variability from the most likely vent position (Fig. 5). For Campi Flegrei, the same combination is less affected by such an underestimation (less than 100 kg/m^2^ difference in the mean tephra load), while the most striking feature is a marked overestimation at proximal distances. These results obtained with the new method are likely due to the fact that, considering the intra-class variability, in the distal areas the new method starts to account for low probability high intensity eruptions with very widespread deposits, whereas in the proximal areas the high probability low intensity eruptions, having the deposits very localised near the vent, become dominant.

When we account for the uncertainty due to the eruptive vent position, *i.e.*, considering any possible vent, in the case of Campi Flegrei, for a fixed explosive eruptive class (Fig. 6, first three rows), we obtain results similar to those obtained for fixed eruptive sizes and vent (Fig. 4). In general, the results obtained accounting for the uncertainty on both the eruptive size and vent position (Fig. 6, bottom row), are not significantly different from those obtained for the combination of simulations from vents fixed at the most likely locations.

These results suggest that, when we consider the combination of all the representative eruptive size classes, the effect of the intra-scenario variability is less important than the inter-scenario variability.

The effect of representative members on the hazard area is similar at the two volcanic systems (right column panels in Figs 3 and 4): the classical method largely overestimates the hazard area for the Small eruptive size class, produces similar results to the new method for the Medium eruptive size class, whereas it tends to underestimate the area for tephra load thresholds lower than 300 kg/m^2^ (a critical one for Neapolitan roofs^33^). Again, this is due to the higher probability associated with eruptive size classes Small and Medium compared to the Large, implying that, when we combine the different size classes, the large overestimation of the hazard area due to the classical method for a small eruption prevails.

It is worth noting that for Medium and Large eruptive size classes, at the critical load threshold of 300 kg/m^2^, both methods produce very similar results (Figs 3 and 4, panels h,i). Moreover, the hazard areas obtained considering the uncertainty on size classes (Fig. 5, panels e,f), are also similar to those calculated for the Medium eruptive size, for both volcanoes (Figs 3 and 4, panels h).

Analysing the results on the hazard area also for different isoprobability contours (10% and 1% in Fig. 7 for the Campi Flegrei case), we observe similar patterns to those found on the area enclosed by the 5% isoprobability contour: while for the Small and Medium size classes the classical method consistently gives larger hazard areas, for the Large size classes there is a crossover tephra load value, depending on the isoprobability contour line, beyond which the new method produces much larger hazard areas (panel j in Fig. 7).

According to the results achieved in this study, the choice of representative members, commonly adopted in volcanic hazard assessment as a way to reduce the computation efforts, is partially justified at proximal-medium distances only, as it tends to produce a hazard assessment that is conservatively higher; however it can significantly underestimate hazard assessment in the distal areas. With respect to the classical method, the new proposed method is able to consider the whole range of values of eruptive parameters that have an influence on tephra dispersal.

By looking at the problem from a risk point of view, results from the two methods are different when one is interested in a specific scenario (fixed size and vent) rather than in an exhaustive combination of scenarios.

As risk assessment often aims at mitigating the effects of a specific hazardous event, this study highlights that the selection of representative scenarios is not univocal, but it depends on the target hazardous event. For example, we have shown that the representative scenarios identified in previous works give opposite results (over- or under-estimates) depending on whether we are considering the effects of tephra fallout for building collapse (*i.e.*, very thick deposits and large tephra load thresholds) or for traffic disruption (*i.e.*, very thin deposits and small tephra load thresholds).

For all these reasons, this study provides a scientific framework for a rational choice of the really most representative eruptive scenarios, which, so far, has always been based on subjective selections, without any rigorous justification.

## Methods

### Modelling approach: the exploration of intra-size variability

In order to carry out tephra fallout simulations, we define three possible eruptive explosive classes^13^,^25^ (respectively for eruptive type and VEI) and characterised by a range of total erupted mass (or magnitudes) in agreement with previous studies (13,26 for Somma-Vesuvius and Campi Flegrei respectively):

1. Small-size class includes small-moderate eruptions^34^ characterised by column heights from 3.5 km to 10 km and a range of total erupted mass of 10^10^–10^11^ kg, hence Magnitude 3 to 4, corresponding to Violent Strombolian type or VEI = 3^13^,^25^. The Small Explosive representative member for Campi Flegrei^16^ and the Violent Strombolian representative member for Somma-Vesuvius^14^ belong to this size class;
2. Medium-size class includes small-moderate to sub-Plinian eruptions^34^ characterised by column heights from 10 km to 20 km and a range of total erupted mass of 10^11^–10^12^ kg, hence Magnitude 4 to 5, corresponding to sub-Plinian I and II type or VEI = 4^13^,^25^. The Medium Explosive representative member for Campi Flegrei^16^ and the sub-Plinian representative member for Somma-Vesuvius^14^ belong to this size class;
3. Large-size class includes sub-Plinian to Plinian eruptions^34^ characterised by column heights from 20 km to 35 km and a range of total erupted mass of 10^12^–10^13^ kg, hence Magnitude 5 to 6, corresponding to Plinian type or VEI ≥ 5^13^,^25^. The Large Explosive representative member for Campi Flegrei^16^ and the Plinian representative member for Somma-Vesuvius^14^ belong to this size class.

For each eruptive size class, we set the PDF for each eruptive parameter (see Table 1, top part), similarly to previous works^35^. The PDF shape and parameters are defined on the basis of previously published papers (5,13,35 for Somma-Vesuvius and^4^,^26^ for Campi Flegrei) in agreement with field observations.

To avoid using representative scenarios, for every simulation we sample a value for the size-related eruptive parameters that are input to the simulators:

1. Sample a value for total erupted mass (or magnitude), duration of the fallout phase, column shape, total grain size distribution and density of tephra particles from their PDFs;
2. Compute the mass fraction *α* associated to tephra fallout with respect to the total erupted mass, where *α* is taken here as 0.8 and 0.25 respectively for Somma-Vesuvius and Campi Flegrei from the available estimations from field data analysis^4^,^13^,^26^;
3. Compute the mean mass eruption rate, using the PDFs listed in Table 1 (top part), in order that the column heights calculated from such mass eruptions rates^36^ range from 3.5 km to 10 km for Small eruptive sizes, from 10 km to 20 km for Medium eruptive sizes, and from 20 km to 35 km for Large eruptive sizes; in this way we obtain mass eruption rates ranging between 2.54 · 10^4^–2.00 · 10^6^ kg/s, 2.00 · 10^6^–3.56 · 10^7^ kg/s and 3.56 · 10^7^–3.62 · 10^8^ kg/s for Small, Medium and Large size class respectively, because the PDF limits were chosen consistently for each eruptive class;
4. Sample a time for the eruption start over a period of 10 years (2001–2010) considering the corresponding meteorological fields for the duration of the fallout phase, and associate this randomly to a combination of the volcanological parameters;
5. Run HAZMAP and FALL3D to obtain the tephra loading at the ground;
6. The probability of each combination is weighted in accord to the associated magnitude.

Aggregation of fine ash is accounted for by a parameterisation^37^. Typical tephra particle densities are chosen consistent with previous values used for Vesuvius^35^ and Campi Flegrei^15^. Total grain size distributions are described as a sum of two log-normal distributions as function of particle diameter choosing distribution parameters in a range close to those describing total grain size distributions reported in previous works on Somma-Vesuvius^14^,^35^,^38^ and Campi Flegrei^15^.

This scheme allows us to explore the variability of eruptive parameters within each eruptive size range identified, rather than assuming a representative scenario for each eruptive size. In other words, the complete range of possible values, for each parameter and within each size class, is sampled in a consistent manner. The added value is given from the inclusion, in the simulations, of “extremely low” or “extremely high” events within each class.

### Total Erupted Mass power law

In order to define a common and continuous probability distribution over the whole total erupted mass range covered by possible eruptions at each of the two volcanoes, we propose a novel methodology. When considering intra-class variability, attention must be paid to appropriately weight events falling within the same size range. While a uniform weight to all the events belonging to a size range would be the most straightforward idea (*e.g.* as in the case of the “eruption range scenario”^39^), it would as well imply a strong dependence on the thresholds dividing class ranges. In fact, under this choice, the largest event of an eruptive size range could have a much larger weight than the smallest event of the subsequent size range. In order to overcome this problem, in this study we first assume a power law (separately at each volcano) on each explosive eruptive size linking the total erupted mass (TEM) within each size class to their observed frequency *N*(TEM) (that will be then generalised to their probability of occurrence):


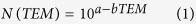


where the total erupted mass spans each of the eruptive size ranges defined above for the Small, Medium, and Large size classes. Such type of power law appears to be an ubiquitous feature characterising the frequency-size relationship of complex natural processes such as earthquakes (the famous Gutenberg-Richter law^21^), landslides, and volcanic eruptions^19^,^20^, among the others. In order to translate the frequency *N*(TEM) into a PDF for the total erupted mass, we impose its integral on the whole TEM domain spanned by explosive eruptions (10^10^−10^13^ kg) to be equal to 1, and we also assume that the slope of the frequency-size relationship (*b*-value) is common among the explosive eruptive size classes (although it can be different between the two volcanoes).

**Figure 2:**
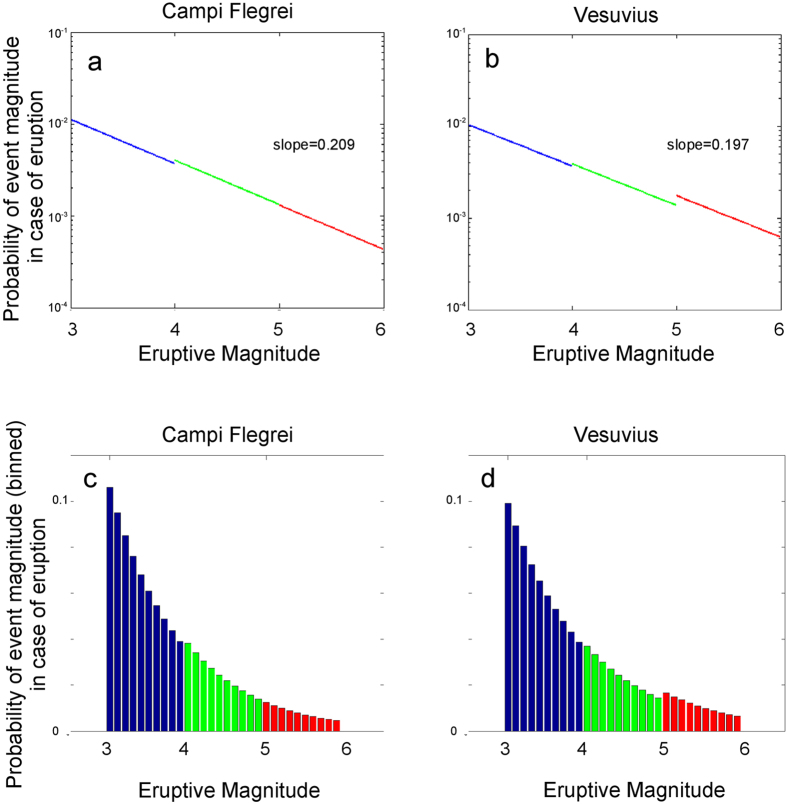
PDF of different eruptive magnitudes in case of an eruption. Panels (**a,b**) report the linear-log resulting relationship, for Campi Flegrei and Somma-Vesuvius respectively (the slope for each volcano is indicated). Panels (**c,d**) show the linear scale histograms when magnitude values are binned into 0.1 units of magnitude. Blue is for the Small size range, green for Medium and red for Large. The area under the blue/green/red part of the plots corresponds to the probability of a Small, Medium and Large size range eruption respectively, conditional to eruption occurrence. These values are in agreement with previous studies for Campi Flegrei^26^ and Somma-Vesuvius^25^

**Figure 3:**
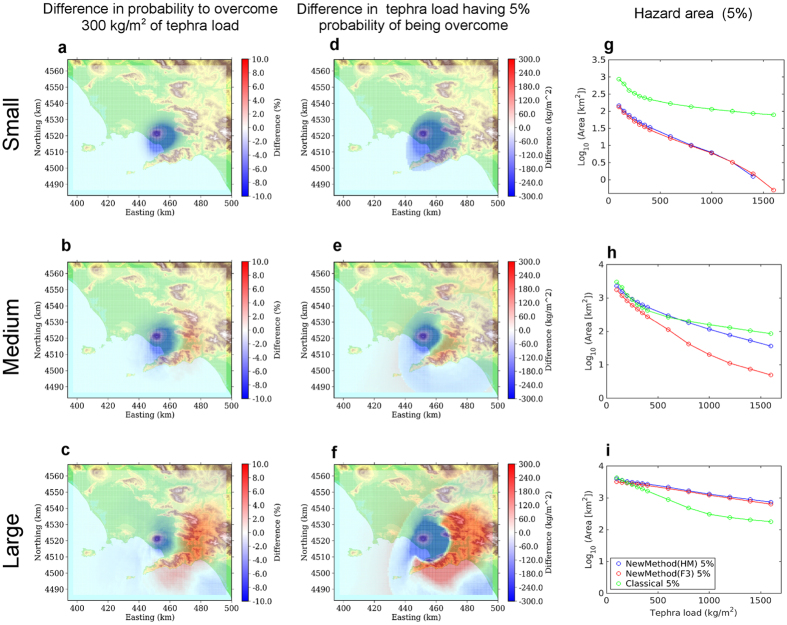
Results for Somma-Vesuvius for Small (top), Medium (middle) and Large (bottom row) size class from summit vent. Left column panels (**a–c**): Difference (given in percent probability) in the probability maps between the new method (according to the simulations by HAZMAP) and the classical method^14^ for a tephra load of 300 kg/m^2^. The probability maps are conditional to the occurrence of an eruption of a specific eruptive size range (Small in panel (**a**), Medium in (**b**) and Large in (**c**)) from the summit vent. Center column panels (**d–f**): Difference (given in tephra load in kg/m^2^) in the hazard maps between the new method (according to the simulations by HAZMAP) and the classical method^14^ for an exceedance probability of 5%. The hazard maps are conditional to the occurrence of an eruption of a specific eruptive size range (Small in panel (**d**), Medium in (**e**) and Large in (**f**)) from the summit vent. In panels (**a**) to (**f**), blue represents an overestimate of the classical model compared to the new, red the opposite. Right column panels (**g–i**): Comparison of the hazard area (Log_10_ of the area in km^2^) enclosed by an isoprobability contour line of 5% of overcoming different tephra load thresholds (on the x-axis). Blue curves are for the new method our model applied to HAZMAP simulations, red for FALL3D and green for the classical method^14^. Northing and Easting coordinates refers to UTM classical system, zone 33T, as in all the following maps. Background maps in this figure have been generated by using the Shuttle Radar Topography Mission (SRTM) data set (90 × 90 m) provided by the CGIAR Consortium for Spatial Information (http://srtm.csi.cgiar.org/) and they have been incorporated in the corresponding panel using Python (https://www.python.org/) software (including Numpy (http://www.numpy.org), and Matplotlib (http://matplotlib.org/) packages); panels have been assembled with LibreOffice (https://www.libreoffice.org/).

**Figure 4:**
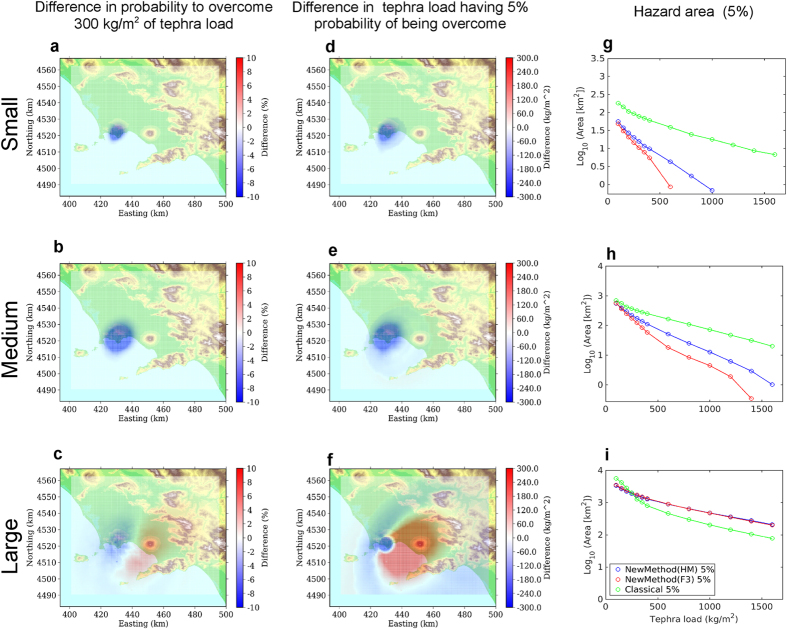
Same as Fig. 3, but for Campi Flegrei eruptions from Astroni vent. Classical method here is ref. 16. Background maps in this figure have been generated by using the Shuttle Radar Topography Mission (SRTM) data set (90 × 90 m) provided by the CGIAR Consortium for Spatial Information (http://srtm.csi.cgiar.org/) and they have been incorporated in the corresponding panel using Python (https://www.python.org/) software (including Numpy (http://www.numpy.org), and Matplotlib (http://matplotlib.org/) packages); panels have been assembled with LibreOffice (https://www.libreoffice.org/).

**Figure 5:**
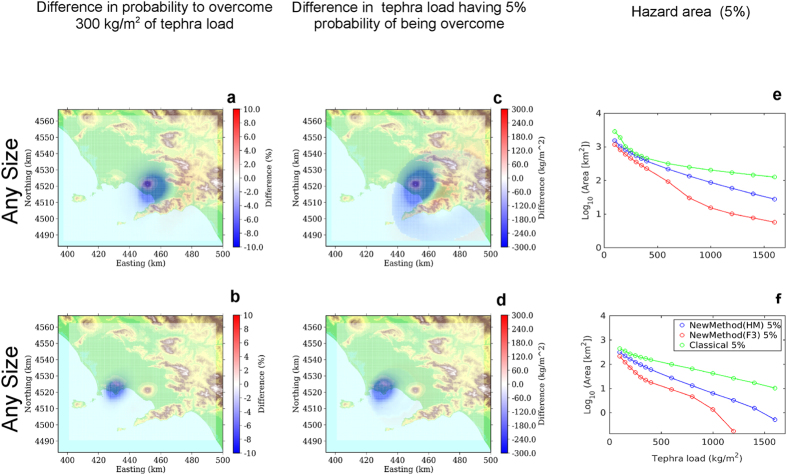
Results obtained when considering the combination of all the possible size classes, from the most likely vent position. In particular, top row is for Somma-Vesuvius (most likely vent position in the summit), and bottom row is for Campi Flegrei (most likely vent position is Astroni). The panels show the same variables as in Fig. 3. Background maps in this figure have been generated by using the Shuttle Radar Topography Mission (SRTM) data set (90 × 90 m) provided by the CGIAR Consortium for Spatial Information (http://srtm.csi.cgiar.org/) and they have been incorporated in the corresponding panel using Python (https://www.python.org/) software (including Numpy (http://www.numpy.org), and Matplotlib (http://matplotlib.org/) packages); panels have been assembled with LibreOffice (https://www.libreoffice.org/).

**Figure 6:**
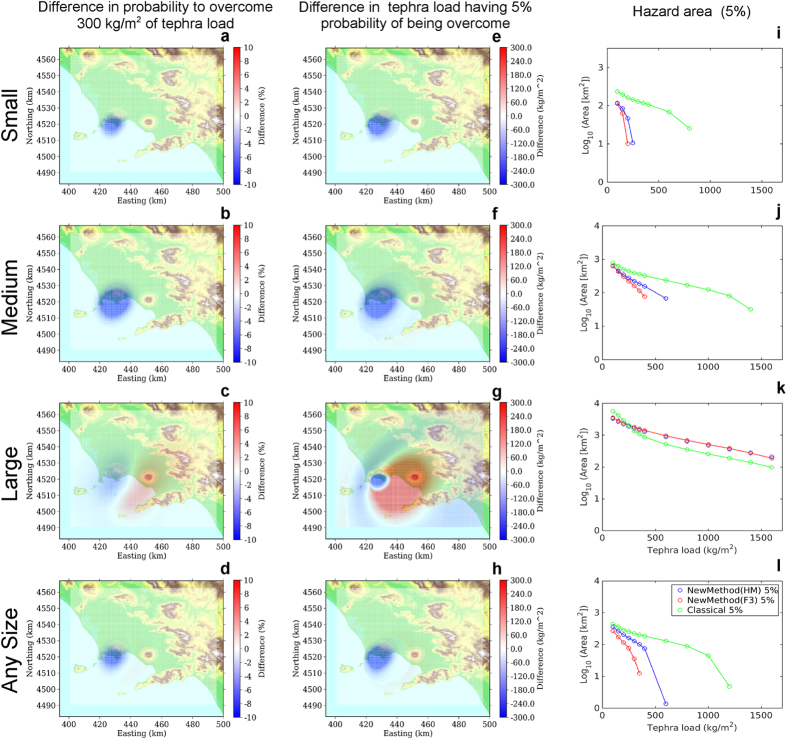
Results for Campi Flegrei when considering uncertainty on vent position: eruptions of Small (top row), Medium (second row), and Large (third row) size class, from any vent. The bottom row shows the results when we consider an eruption of any size class, from any vent. The panels show the same variables as in Fig. 3. Background maps in this figure have been generated by using the Shuttle Radar Topography Mission (SRTM) data set (90 × 90 m) provided by the CGIAR Consortium for Spatial Information (http://srtm.csi.cgiar.org/) and they have been incorporated in the corresponding panel using Python (https://www.python.org/) software (including Numpy (http://www.numpy.org), and Matplotlib (http://matplotlib.org/) packages); panels have been assembled with LibreOffice (https://www.libreoffice.org/).

**Figure 7:**
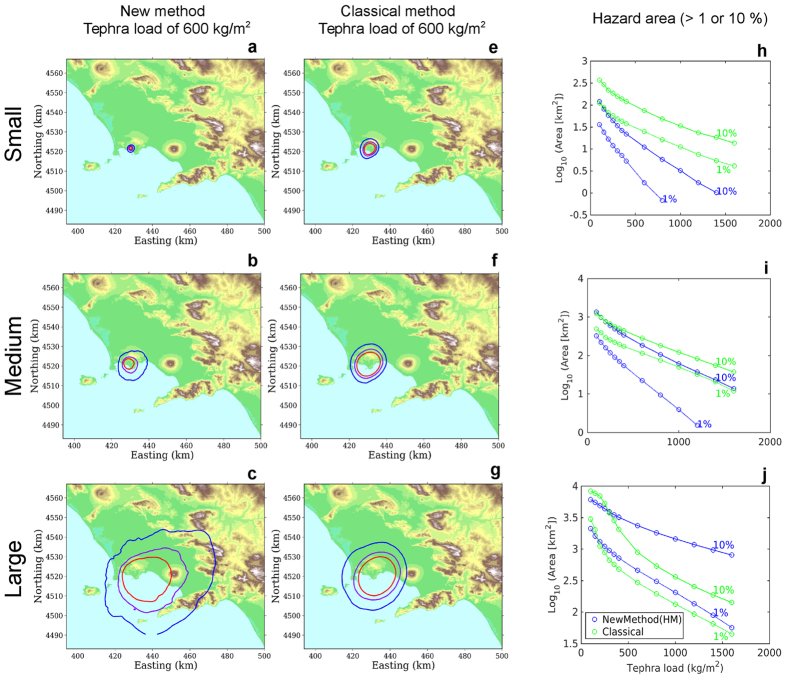
Comparison of the hazard area (Log_10_ of the area in km^2^) enclosed by different isoprobability contour lines for a Campi Flegrei eruption from Astroni vent (Small, Medium and Large size classes from top to bottom). In the maps, we plot contour lines enclosing the area with a probability of 10% (red line), 5% (purple line) and 1% (blue line) of overcoming a 600 kg/m^2^ tephra load threshold, as resulting from the new method with HAZMAP simulations (**a–c**), and from the classical method (**d–f**). In the line plots, we show the area enclosed by isoprobability contour lines of 10% (solid) and 1% (dashed lines), of overcoming different tephra load thresholds (on the x-axis). Blue is for the new method applied to HAZMAP simulations, and green for the classical method^16^. Background maps in this figure have been generated by using the Shuttle Radar Topography Mission (SRTM) data set (90 × 90 m) provided by the CGIAR Consortium for Spatial Information (http://srtm.csi.cgiar.org/) and they have been incorporated in the corresponding panel using Python (https://www.python.org/) software (including Numpy (http://www.numpy.org), and Matplotlib (http://matplotlib.org/) packages); panels have been assembled with LibreOffice (https://www.libreoffice.org/).

A Bayesian inferential method has previously been proposed^25^ to determine a Dirichlet PDF describing the probability of three random events, *i.e.*, a VEI = 3, VEI = 4 and VEI = 5+ eruption at Somma-Vesuvius, given the occurrence of an eruption. According to^25^, these random events represent a set of mutually exclusive and collectively exhaustive outcomes, given an eruption at vesuvius, and thus the assumption of a Dirichlet PDF is justified. Similarly, at Campi Flegrei a Dirichlet PDF has also been proposed^26^ to describe the probability of four mutually exclusive and collectively exhaustive random events, *i.e.*, the occurrence of an effusive, small-, medium- and large-explosive event, conditional to the occurrence of an eruption. Both methods assume a prior power law; further, they both make use of the number of events with different eruptive sizes observed over a complete portion of the volcano eruptive catalogue, to build a likelihood function. The parameters of the resulting posterior PDF obtained in these studies are here reported in Table 1 (bottom part), for Campi Flegrei and Somma-Vesuvius (although for Campi Flegrei, the counting of Large size events has been here revised to 2). Such parameters can be interpreted as the expected number of “successes” for every possible event described by the PDF. In our case, this translates into the expected number of eruptions of the different sizes, for each of the two volcanic systems. Because of this, we use the posterior Dirichlet parameters reported in Table 1 (bottom part) as a fictitious sample of observed eruptions at the two volcanoes, and determine the common *b*-value of eq.1 through the maximum likelihood method^40^. This ensures that the final distribution is in agreement with the results by the latter studies in terms of relative mean probabilities of the corresponding Small, Medium and Large size classes at the two volcanoes, conditional to the occurrence of an eruption. Simultaneously, it allows for determining a PDF over the whole TEM range, characterised by a common *b*-value, that is here used to determine the PDF of every possible TEM value (see Fig. 2).

### Simulation setup

For each volcano (Somma-Vesuvius and Campi Flegrei) and eruptive size range (S, M and L), we random sample 1500 wind fields on the time interval 2001–2010 from ECMWF Reanalysis database. Then, we run 1500 simulations with HAZMAP and FALL3D separately, in each randomly combining meteorological conditions and volcanological parameters (as from the scheme in section).

The simulation scheme (1500 sampling and consequent simulations for each eruptive size class and each volcano) is run on a rectangular domain encompassed between 40.50N and 41.25N (on latitude) and 13.75E and 15.00E (on longitude). Original eruptive vent is simulated at (40.835N 14.166E) and (40.821N 14.426E) respectively for Campi Flegrei and Somma-Vesuvius. The simulation grid is 500 m spaced for HAZMAP and 0.015 degrees (≈1.5 km) for FALL3D. The coarser grid for the latter simulator is necessary for keeping the computational time within reasonable limits (about 6500 run-time hours with 30–32 CPUs available on the Barcelona Supercomputing Centre cluster). The output of FALL3D simulation is then interpolated on the finer HAZMAP grid.

## Additional Information

**How to cite this article**: Sandri, L. *et al.* Beyond eruptive scenarios: assessing tephra fallout hazard from Neapolitan volcanoes. *Sci. Rep.* **6**, 24271; doi: 10.1038/srep24271 (2016).

## Supplementary Material

Supplementary Information

## Figures and Tables

**Table 1:** Top part: PDFs for the main eruptive parameters for Somma-Vesuvius and Campi Flegrei.

Parameter	Eruption Size	PDF type and parameters	Classical method
Total erupted mass (kg)^*a*^	Small Somma-Vesuvius	Uniform on [10^10^; 10^11^]	2.5.10^11^
	Small Campi Flegrei	Uniform on [10^10^; 10^11^]	9.2.10^10^
	Medium Somma-Vesuvius	Uniform on [10^11^; 10^12^]	6.3.10^11^
	Medium Campi Flegrei	Uniform on [10^11^; 10^12^]	4.8.10^11^
	Large Somma-Vesuvius	Uniform on [10^12^; 10^13^]	2.5.10^12^
	Large Campi Flegrei	Uniform on [10^12^; 10^13^]	2.1.10^12^
Duration of fallout phase (hours)	Small Somma-Vesuvius	Uniform on [11.11; 87.60]	≈110
	Small Campi Flegrei	Uniform on [3.48; 27.36]	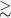 6.4
	Medium Somma-Vesuvius	Uniform on [6.24; 11.11]	≈4.5
	Medium Campi Flegrei	Uniform on [1.95; 3.48]	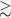 3.3
	Large Somma-Vesuvius	Uniform on [6.14; 6.24]	≈7
	Large Campi Flegrei	Uniform on [1.92; 1.95]	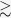 1.4
Mass Eruption Rate (kg/s)	Small Somma-Vesuvius	[1.5 ⋅ 10^6^, 1.2 ⋅ 10^8^]	≈5 ⋅ 10^5^
	Small Campi Flegrei	[1.5 ⋅ 10^6^, 1.2 ⋅ 10^8^]	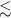 10^6^
	Medium Somma-Vesuvius	[1.2 ⋅ 10^8^, 2.1 ⋅ 10^9^]	≈3.10^7^
	Medium Campi Flegrei	[1.2 ⋅ 10^8^, 2.1 ⋅ 10^9^]	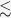 10^7^
	Large Somma-Vesuvius	[2.1 ⋅ 10^9^, 2.2 ⋅ 10^10^]	≈8.10^7^
	Large Campi Flegrei	[2.1 ⋅ 10^9^, 2.2 ⋅ 10^10^]	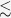 10^8^
Total Grain Size Distribution modes (Φ − *units*)	Small Somma-Vesuvius	Beta on [−3.0; 0.0] for *μ*_*c*_ and [1.5; 3.5] for *μ*_*f*_	Macedonio *et al.*^14^
	Small Campi Flegrei	Beta on [−2; 0.5] for *μ*_*c*_ and [3.5; 5.5] for *μ*_*f*_	Costa *et al.*^15^
	Medium Somma-Vesuvius	Beta on [−1.0; 3.0] for *μ*_*c*_ and [4.5; 6.5] for *μ*_*f*_	Macedonio *et al.*^14^
	Medium Campi Flegrei	Beta on [−3.5; 0.5] for *μ*_*c*_ and [3.5; 5.5] for *μ*_*f*_	Costa *et al.*^15^
	Large Somma-Vesuvius	Beta on [−1.0; 3.0] for *μ*_*c*_ and [4.5; 6.5] for *μ*_*f*_	Macedonio *et al.*^14^
	Large Campi Flegrei	Beta on [−3.5; 0.5] for *μ*_*c*_ and [3.5; 5.5] for *μ*_*f*_	Costa *et al.*^15^
Density of tephra particles (kg/m^3^)	Somma-Vesuvius (any size)	Beta on [900; 1600] for *ρ*_*c*_ and [2500; 2900] for *ρ*_*f*_	Macedonio *et al.*^14^
	Campi Flegrei (any size)	Beta on [900; 1600] for *ρ*_*c*_ and [2500; 2900] for *ρ*_*f*_	Costa *et al.*^15^
**Volcano**	**Size**	**Corresponding Dirichlet Parameter**	**Mean probability**
Somma-Vesuvius	VEI = 3 (Small)	6.49	0.65
	VEI = 4 (Medium)	2.42	0.24
	VEI = 5+ (Large)	1.09	0.11
Campi Flegrei	Effusive	3.20	0.11
	Small-Explosive (Small)	18.2	0.60
	Medium-Explosive (Medium)	6.52	0.22
	Large-Explosive (Large)	2.12	0.07

Bounds on Mass Eruption Rate values are a consequence of the stratified sampling procedure for total erupted mass and duration of the fallout phase described in the text. For the total grain size distribution, *μ*_*c*_ and *μ*_*f*_ are respectively the modes of coarse and fine particles. For the density of tephra particles, *ρ*_*c*_ and *ρ*_*f*_ are respectively the density of coarse and fine particles. For the data on the other input parameters, see Supplementary Table S1. ^α^considering an average density of about 10^3^ kg/m^3^, these values imply erupted volume ranges of 0.01–0.1, 0.1–1 and 1–10 km^3^ respectively for Small, Medium, and Large size range. These are not in complete agreement with what proposed by^13^ who identified 4 scenarios at Somma-Vesuvius. With respect to^13^, we use a simpler division and neglect Violent Strombolian with very small volume (0.001 km^3^). However, such type produces very thin deposits. In the rightmost column we give (where possible) the corresponding values adopted in the classical method studies (14 for Somma-Vesuvius, 16 for Campi Flegrei) that we use for comparison. Bottom part: Values of the Dirichlet distribution’s parameters for the various eruptive sizes for Somma-Vesuvius^25^ and Campi Flegrei^26^. For Campi Flegrei, the values for the Large size have been updated by dividing the Agnano-MonteSpina event into two separate events.
