# Supplementary material for: Beyond eruptive scenarios: assessing tephra fallout hazard from Neapolitan volcanoes
Source: Sci Rep. 2016 Apr 12;6:24271. doi: 10.1038/srep24271 (PMC4828649; doi:10.1038/srep24271)
Supplement: Supplementary Information [file srep24271-s1.pdf]

Beyond eruptive scenarios: assessing tephra fallout hazard from Neapolitan volcanoes

Laura Sandri*, Antonio Costa, Jacopo Selva, Roberto Tonini,
Giovanni Macedonio, Arnau Folch, Roberto Sulpizio

* *laura.sandri@ingv.it*

Supplementary Online Information

Table S1

Eruption Size	σ_f ($\Phi - units$)	σ_c ($\Phi - units$)	p	ψ	A
Small					
SV	1.7	1.5	0.5	Uniform on [0.90. 0.94]	Beta on [2.5 ; 4.5]
CF	1.19	1.19	0.2	Uniform on [0.90. 0.94]	Beta on [2.5 ; 4.5]
Medium					
SV	2.3	1.5	0.75	Uniform on [0.90. 0.94]	Beta on [3.0 ; 4.5]
CF	1.54	1.54	0.3	Uniform on [0.90. 0.94]	Beta on [3.0 ; 4.5]
Large					
SV	2.3	1.5	0.75	Uniform on [0.90. 0.94]	Beta on [3.5 ; 4.5]
CF	1.38	1.38	0.4	Uniform on [0.90. 0.94]	Beta on [3.5 ; 4.5]

TABLE S1: Input parameters to numerical models HAZMAP and FALL3D for simulating tephra transport and dispersal with the new method. SV is Somma-Vesuvius, CF is Campi Flegrei. σ_c and σ_f refer to the standard deviation of the Normal distributions associated to the total grain size distribution, respectively for coarse and fine particles. The parameter p is the relative weight of the coarse particles in the total grain size distribution. The parameter ψ is the particle sphericity, assuming a symmetrical beta distribution. The parameter A is Suzuki (1983) column shape parameter.

Suzuki, T., 1983. A theoretical model for dispersion of tephra. In: Shimozuru, D., Yokoyama, I. (Eds.), Arc Volcanism: Physics and Tectonics. Terra Scientific Publish- ing Company (TERRAPUB), Tokyo, pp. 93113.
